# Distinct roles of claws and adhesive pads in honey bees determine their adhesive performance under varying surface roughness conditions through a dual-mode synergistic adhesion mechanism

**DOI:** 10.1371/journal.pone.0329624

**Published:** 2025-08-04

**Authors:** Youhe Yu, Hongqi Luo, Yongxia Gu, Jieliang Zhao

**Affiliations:** 1 School of Computer and Artificial Intelligence, Beijing Technology and Business University, Beijing, China; 2 School of Mechanical Engineering, Beijing Institute of Technology, Beijing, China; University of Life Sciences in Lublin, POLAND

## Abstract

Surface roughness is a critical factor that affects surface adhesion in bees. Investigating the mechanisms underlying surface adhesion in bees on substrates with varying surface roughness levels provides a theoretical basis for designing bioinspired adhesives and micro-climbing robots. In this study, a specialized adhesion measurement device was developed to compare the adhesive forces applied on substrates with different roughness levels by intact bees and by those whose adhesive pads have been removed. Moreover, a contact model for adhesion between the claw tips and substrate particles and a liquid bridge model for adhesion between the adhesive pads and substrate surfaces were established to analyze the distinct roles of claws and adhesive pads, respectively, under different surface roughness conditions. The results revealed that on surfaces with roughness values below 3.2 μm, the adhesive pads secreted liquid to form liquid bridges, increasing contact area and adhesive force, thereby dominating the adhesion process. As the surface roughness increased, the contribution of the adhesive pads diminished, whereas the mechanical interlocking effect of the claws became relatively more pronounced. When the surface roughness exceeded 36 μm, the irregularity of substrate particles enhanced the interlocking effect of the claws, making the force generated by the claw tip the dominant adhesion force. Thus, the integration of the contact and liquid bridge models revealed the synergistic effects of mechanical interlocking and liquid bridge mechanisms on improvement in surface adhesion in bees.

## Introduction

The adhesion mechanisms of insects have become a focal point of research in biomimetics and biomechanics owing to their remarkable adaptability to complex surfaces. This synergistic interaction primarily mediates the adhesive behavior between the claws and adhesive pads. In wet-adhesion insects, the adhesive pads secrete mucus to form liquid bridges, enhancing capillary and adhesive forces, whereas the claws engage in mechanical interlocking with surface irregularities, creating a dual-adhesion strategy [1,2]. This bimodal mechanism provides significant insight into the design of bioinspired adhesives [3] and micro-climbing robots [4]. Cross-species studies on insect adhesion have shown that different taxa exhibit significant variation in their adaptability to surface roughness. For example, ants demonstrate the highest attachment strength on surfaces with a roughness value of approximately 5 μm, with the elastic deformation of their adhesive pads dynamically adapting to micron-scale surface undulations [5,6]. In contrast, the claw-pad synergy in beetles dominates on rough surfaces (Ra > 50 μm), where the ratio of the claw tip diameter to particle size determines the mechanical interlocking efficiency [7]. These studies have primarily focused on extremely rough surfaces (smooth or highly rough), and there remains a lack of a comprehensive analysis of surface roughness in the range of 3–30 μm.

Persson and Gorb proposed the elastic contact theory wherein liquid bridges increase the contact area by filling micro-gaps on surfaces with low roughness, whereas mechanical interlocking enhances friction on surfaces with relatively high roughness through geometric engagement [8]. This theory has been validated in studies on the tarsal pads of cockroaches (*Periplaneta americana*). However, the model did not quantify the impact of the liquid bridge volume on the adhesive forces [9]. Chen et al. discovered through nanoscale liquid bridge experiments that the surface roughness significantly influences the rupture distance of liquid bridges [10]. The adhesion performance is strongly influenced by the multiscale characteristics of the surface topography. For instance, nanoscale surface roughness (*Ra* < 100 nm) enhances adhesion stability by increasing the rupture energy of liquid bridges [11], whereas micron-scale undulations (*Ra* = 1–10 μm) reduce capillary forces by limiting the extension of liquid bridges [12]. These findings highlight the complex nonlinear relationship between surface roughness and adhesion mechanisms. Existing models struggle to quantify the coupling effects of multiscale topography and dynamic environments [13].

Surface roughness is a critical factor that affects surface adhesion in bees. Investigating the mechanisms underlying surface adhesion in bees on substrates with varying surface roughness levels provides a theoretical basis for designing bioinspired adhesives and micro-climbing robots. The primary aim of this study is to elucidate the distinct roles of claws and adhesive pads in honey bees and how they contribute to the overall adhesive performance under different surface roughness conditions. Specifically, we aim to:

1. Compare the adhesive forces of intact honey bees (with both claws and adhesive pads) and bees with removed adhesive pads on surfaces with different roughness levels.
2. Establish contact and liquid bridge models to analyze the individual contributions of claws and adhesive pads to surface adhesion.
3. Reveal the synergistic effects of mechanical interlocking (via claws) and liquid bridge formation (via adhesive pads) on enhancing adhesion under different surface roughness conditions.

In this study, we developed a specialized adhesion measurement device to compare the adhesive forces exerted by intact bees and bees with removed adhesive pads on substrates with varying surface roughness levels. We also established contact and liquid bridge models to elucidate the distinct contributions of claws and adhesive pads under different surface roughness conditions. Our results will provide a comprehensive understanding of the dual-mode synergistic adhesion mechanism of bees on surfaces with different roughness levels and offer theoretical support for the development of bioinspired adhesives suitable for diverse surface conditions.

## Materials and methods

### Experimental animals

Adult Italian honey bees (*Apis mellifera ligustica*) were obtained from the Xiangshan Apiary in Beijing, China. The bees were maintained in a transparent glass chamber sealed on all sides with a ventilation port at the top, under controlled laboratory conditions at 25°C and 50% relative humidity. They were fed a mixture of water and honey in a 1:1 (v/v) ratio as their food source. All procedures followed the national standards for animal welfare in China (GB/39760–2021) and European Directive 2010/63/EU on the protection of animals used for scientific purposes. Since the bees used in this study were purchased through commercial channels and all experimental activities were conducted within a laboratory environment without field collection or interference with natural ecosystems, no fieldwork permit was required for this study.

**Fig 1:**
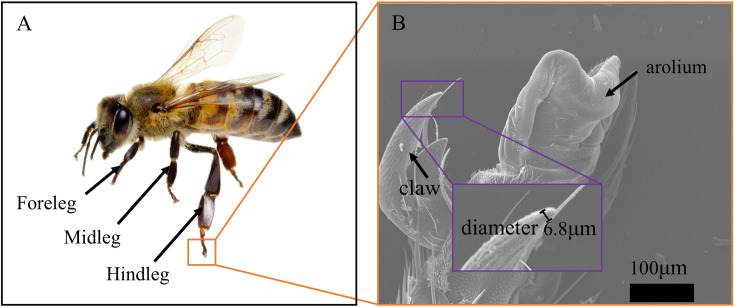
Animal used in the experiment. **(A)** The right hind leg of the honey bee was chosen for the experiment. **(B)** Scanning electron microscopy image of the tip of the claw of a honey bee, showing a diameter of 6.8 μm.

To investigate the combined effect of adhesive pads and claws, bees were divided into two groups: intact bees (with adhesive pads) and bees with adhesive pads removed (without adhesive pads). The hind legs of relatively large bees were selected as experimental subjects. Intact bees (with adhesive pads) refer to bees that have not undergone any surgical procedures. Under a stereomicroscope, fine microsurgical scissors were used to surgically excise the adhesive pads while preserving the claws and other tarsal structures, thereby obtaining bees without adhesive pads. After the surgery, 95% ethanol was applied to the wound site. The bees were then allowed to recover at room temperature for 15–20 minutes. Adhesion performance testing was conducted once the hemolymph no longer leaked from the wound. The adhesive force attributable to the bee’s adhesive pads alone can be determined by calculating the difference in mean values between intact bees and bees from which the adhesive pads have been removed. Each group consisted of 10 bees, with an average weight of 128.90 ± 13.71 mg and an average side claw tip diameter of 6.8 μm (Fig 1). During the weighing process, the bees exhibited considerable activity, which may have introduced variability in the measured weights. This activity could account for the observed average weight of 128.90 ± 13.71 mg, which is slightly higher than the commonly reported average weight of approximately 100 mg for honey bees. Despite this potential source of error, the weight of individual bees did not significantly influence the adhesion force measurements, as the bees were inverted and secured in a custom-designed holder during the testing process, with their weight effectively counteracted by the holding apparatus. The adhesion force measurements were conducted on the right hind legs of the bees in multiple trials.

### Experimental substrates

To investigate the effects of surface roughness on surface adhesion in bees, smooth glass and seven different grades of sandpaper (ranging from 1,000–20,000 grit; ADZ, Japan) were used as substrates. All sandpapers had the same material composition and surface characteristics. The sandpaper was then rinsed with deionized water and dried to remove impurities from the surface. Surface roughness parameters, including *Ra* (arithmetic mean surface roughness), *Rq* (root mean square roughness), and *Rz* (maximum profile height), were measured using a laser scanning confocal microscope (N-SIM E, Nikon, Japan). Measurements were taken within a 2 mm × 2 mm square area with a sampling speed of 6.67 mm/s and a frequency of 2000 Hz. This allowed for the precise characterization of the surface topography of each substrate to ensure consistency in the roughness levels across different materials.

The measured substrate surface particle diameters ranged from 1.6 μm to 268.6 μm, covering a broad range of roughness levels, which is conducive to a comprehensive evaluation of adhesion forces under different surface roughness conditions. The average diameters of the surface particles were used to represent the different roughness levels. The specific roughness values of the substrates are listed in Table 1.

**Table 1:** Parameters determining the surface roughness of sandpapers.

Type of sandpaper	*Ra* (μm)	*Rq* (μm)	*Rz* (μm)	Mean diameter (μm)
20,000 grit	3.79	2.88	27.5	1.6
10,000 grit	3.98	4.8	36.5	3.2
8,000 grit	4.91	6.23	56.3	5
6,000 grit	7.02	13.4	69	6.4
4,000 grit	19.3	74.3	219	36
2,000 grit	85	289	980	167
1,000 grit	138	316	1611	268.6

^a^*Ra*, arithmetic mean surface roughness; *Rq*, root mean square roughness; and *Rz*, maximum profile height.

### Adhesion force measurement

**Fig 2:**
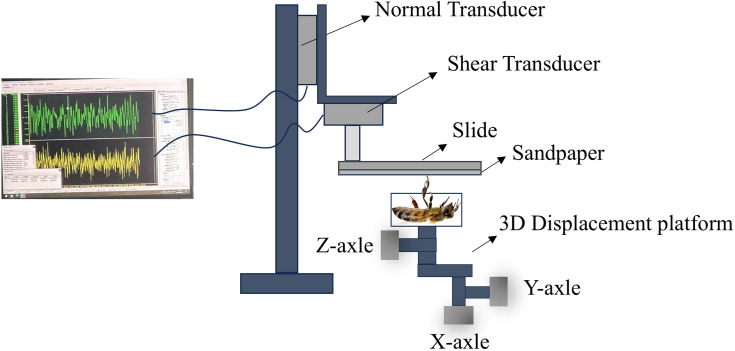
Adhesion measurement device developed in this study.

The adhesion forces on substrates with different surface roughness levels were measured using high-precision biaxial force sensors (models GSO-10 and GSO-50; Transducer Techniques, USA). The sensor has a measurement range of 100 mN for normal force and 500 mN for shear force, with a resolution of 0.1 mN and a data acquisition frequency of 1 kHz. The force sensor was mounted on a motorized platform that moved at a constant speed of 5 mm/s along the *Z*-axle (normal direction) and *X*-axle (shear direction), as illustrated in Fig 2. This setup enabled the precise measurement of both normal and shear forces during the detachment process, ensuring that the contributions of both the types of forces to the overall adhesion could be accurately quantified across different surface roughness conditions.

During the experiment, a heat-resistant adhesive was used to fix the sandpaper onto a rigid platform to ensure the stability of the substrate during the measurements. The bees were secured in a custom-designed 3D-printed holder with transparent tape gently restricting their body while allowing free movement of the hind legs. The force sensor was activated during the experiment. As one of the bee’s hind legs was left unfixed, the bee could freely perform attachment and detachment movements. The maximum detachment force recorded when the adhesive pad or claw contacted the substrate was documented as the adhesive force. Each bee was subjected to three repeated measurements on each substrate. The adhesion force results were expressed as the mean ± standard deviation (Mean ± SD), and statistical significance between groups was assessed using independent sample *t*-tests and multiple comparisons. The data on normal and shear adhesive forces measured for intact bees and bees with adhesive pads removed on surfaces with different roughness levels are provided in S1 File.

## Results

### Normal adhesion force

**Fig 3:**
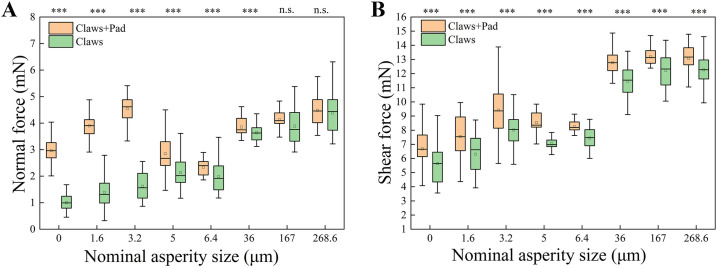
Forces applied by honey bees on different surfaces. **(A)** Box plots of normal force generated by honey bees on surfaces of varying roughness presenting a comparative analysis of the forces generated by honey bees with intact adhesive pads (orange) and those with only claws (green). **(B)** Box plots of shear force generated by honey bees on surfaces of varying roughness presenting a comparison of the forces generated by honey bees with intact adhesive pads (orange) and those with only claws (green). Statistically significant differences are indicated by ***P < 0.001, **P < 0.01, and *P < 0.05, and n.s. denotes statistically non-significant differences.

The measurement results of the normal adhesion force (Fig 3A) showed significant differences between intact bees and bees without adhesive pads on substrates with an average particle diameter of ≤ 36 μm. For intact bees, the normal adhesion force reached a maximum value of 5.1 mN on the 3.2 μm surface. However, as the roughness increased to 6.4 μm, the normal adhesion force decreased to 2.0 mN. Bees without adhesive pads exhibited lower normal adhesion forces on surfaces with lower roughness values. On smooth surfaces, as well as on surfaces with 1.6 μm and 3.2 μm roughness, the normal adhesion force decreased by 66.3%, 66.4%, and 64.3%, respectively, compared to the intact bees. On surfaces with 5 μm and 6.4 μm roughness, the adhesion forces were 2.1 mN and 2.0 mN, showing slight variation.

As the surface roughness increased (≥ 36 μm), the average normal adhesion force for both groups increased. On surfaces with roughness values ranging from 36 μm to 268.6 μm, the normal adhesion forces for intact bees were 3.9 mN, 4.1 mN, and 4.5 mN, respectively, while for bees with the adhesive pads removed, the normal adhesion force decreased by only 5.7%, 5.7%, and 2.2%, respectively. On surfaces with roughness values of 167 μm and 268.6 μm, there were no significant differences in the measured results between the two groups (at 167 μm, *T* = 1.669, **P* *= 0.101; at 268 μm, *T* = 0.588, *P* = 0.559).

The magnitude of the normal force is primarily influenced by the combined effects of the liquid bridge and mechanical interlocking. In the experiment, the comparison between intact bees and bees with removed adhesive pads offers crucial information, helping to indirectly uncover the adhesive performance of the pads alone. On smooth surfaces, bees without adhesive pads show a marked reduction in normal force due to the inability to form effective liquid bridges or mechanical interlocking. When intact bees are contrasted with those that have had their adhesive pads removed, the lower normal force observed in the latter indicates that the liquid bridge plays a vital role in generating normal force on smooth surfaces. Similar conclusions were drawn by Prokkola et al. in their study of cockroach adhesive pads [9], where they posited that on smooth surfaces, the liquid bridge effect is dominant, and the lack of mechanical interlocking leads to a significant decrease in adhesion.

On surfaces with 1.6μm and 3.2μm roughness, the normal force of bees without adhesive pads increased. The moderate particle size allowed the lateral claws to better embed into the rough surface, enhancing mechanical interlocking. However, compared to intact bees, their normal force remained lower, highlighting the liquid bridge effect’s crucial role in boosting adhesion even when mechanical interlocking is enhanced. Zhu et al. showed that at moderate roughness, the liquid bridge effect maximizes adhesion by increasing contact area and capillary forces, aligning with the observations in intact bee experiments and underscoring the liquid bridge effect’s significance on surfaces with low roughness [14].

As the roughness increased to 5–6.4μm, the normal force of intact bees began to decrease. This indicates that the liquid bridge effect was weakened, as the adhesive fluid couldn’t fully fill the larger gaps between particles, thus reducing adhesion. A similar observation was made in ant adhesion studies. Federle et al. pointed out that on rough surfaces, when the liquid bridge effect is weakened, adhesion drops significantly, and mechanical interlocking starts to dominate the adhesion process [15]. On surfaces with even higher roughness (36–268.6μm), the normal force of bees, whether they had their adhesive pads removed or not, increased considerably. This is mainly because the roughness particles were much larger than the claw tip diameter, significantly enhancing the mechanical interlocking effect and making it the dominant factor for adhesion. This is in line with findings on mechanical interlocking in bionics research, especially in studies of biomimetic soft robotic footpad systems. Dang et al. demonstrated that on such rough surfaces, the engagement between claw tips and particles generates a strong normal force [16].

### Shear adhesion force

The results of the shear adhesion force measurements (Fig 3B) revealed significant differences between the shear forces applied on substrates with different surface roughness levels by intact bees and by bees with their adhesive pads removed. In both groups, the shear adhesion force increased gradually as the surface roughness increased from smooth surfaces to 3.2 μm. The shear adhesion forces for intact bees were 6.7 mN, 7.6 mN, and 9.4 mN, peaking at 3.2 μm. In comparison, the shear adhesion force for bees with adhesive pads removed decreased by 15.7%, 16.6%, and 14.9%, respectively, compared with intact bees. However, as the surface roughness increased further, the shear adhesion forces for both groups of bees decreased.

On relatively rough surfaces (36 μm to 268.6 μm), the shear adhesion force for both groups significantly increased. On the 36 μm surface, the shear adhesion force for intact bees reached 12.8 mN, while bees with the adhesive pads removed reached 11.5 mN. At 167 μm, the maximum shear adhesion force for intact bees was recorded at 13.7 mN, while bees with the adhesive pads removed reached a maximum of 13.5 mN at 268.6 μm. On surfaces ranging from 36 μm to 268.6 μm, the shear adhesion force for bees without adhesive pads was reduced by 10.3%, 7.3%, and 6.3%, respectively, compared to the intact bees.

The data were analyzed using OriginPro 2021 software, and the measurement results satisfied a normal distribution. Independent-sample *t*-tests and Welch’s *t*-tests (for unequal variances) were used to compare the adhesion forces between intact bees and bees after the adhesive pads were removed. One-way analysis of variance (ANOVA) and Bonferroni correction were applied for multiple comparisons across the roughness levels. Statistical significance was set at *P* < 0.05, and the results were presented as means. Box plots were used to visualize the distribution of the normal and shear adhesion forces, highlighting the differences between the groups and roughness levels. Table 2 presents the results of independent *t*-*t*ests.

**Table 2:** Summary of the results of independent *t*-tests.

Nominal asperity size(μm)	Normal forces		Friction forces	
Smooth	*T* = 19.074	*P* = 0.000***	*T* = 2.757	*P* = 0.008***
1.6	*T* = 19.102	*P* = 0.000***	*T* = 3.308	*P* = 0.002***
3.2	*T* = 21.753	*P* = 0.000***	*T* = 3.476	*P* = 0.001***
5	*T* = 4.296	*P* = 0.000***	*T* = 10.383	*P* = 0.000***
6.4	*T* = 2.82	*P* = 0.007***	*T* = 5.28	*P* = 0.000***
36	*T* = 2.424	*P* = 0.000***	*T* = 5.099	*P* = 0.000***
167	*T* = 1.669	*P* = 0.101	*T* = 4.139	*P* = 0.000***
268.6	*T* = 0.588	*P* = 0.559	*T* = 3.079	*P* = 0.003***

^a^This table presents the *T*-values and *P*-values obtained from independent sample *t*-tests, comparing the distribution of the normal forces generated by honey bees with and without adhesive pads across surfaces of varying roughness. The *T*-value reflects the significance of the difference in the means between the two groups. *P*-values indicate the statistical significance of these differences, with ***P < 0.001, **P < 0.01, and *P < 0.05 considered statistically significant. Statistically non-significant differences (*P* > 0.05) are marked as n.s. (not significant).

## Discussion

### Mechanical interlocking model of contact between claw tip and sandpaper particle

The interaction between the honey bee claw tip and sandpaper particles is crucial for adhesion, particularly on rough surfaces. When the substrate surface particle diameter is smaller than the claw tip diameter, mechanical interlocking fails, causing slippage and weaker adhesion. In contrast, when the particle diameter of the substrate surface significantly exceeds the claw tip diameter, the claw tip can effectively engage with the irregularities of the sandpaper, forming mechanical interlocking, thereby enhancing adhesion.

**Fig 4:**
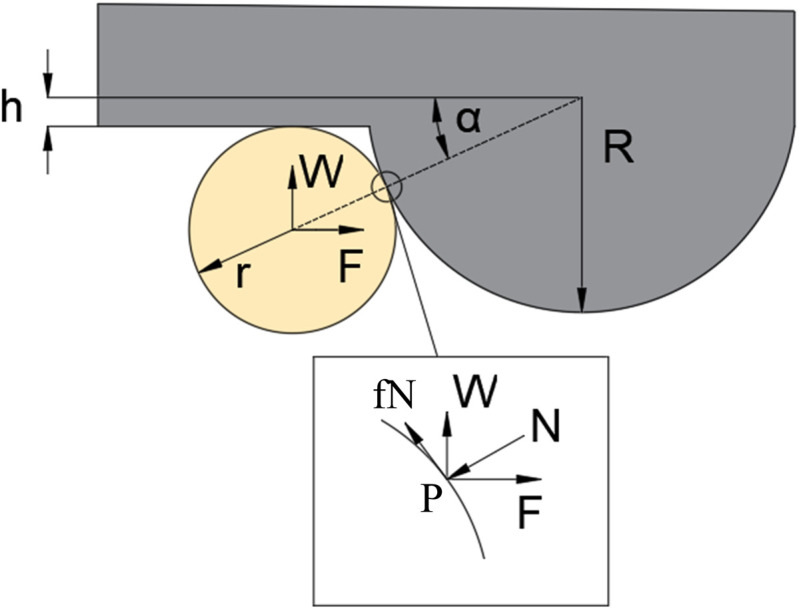
Model for the interaction of the claw tip of a honey bee with surface irregularities. The gray bumps represent rough basal particles, and the yellow spherical represent the tips of the claws. α, the contact angle, is the angle between the line connecting the centers of the hemispherical particle and the claw tip and the horizontal direction; F, the tangential force generated by the bee’s leg; fN, line running perpendicular to the normal line N running through both centers of the particle and the claw tip; h, the depth of the hemispherical particle embedded in the substrate; r, the claw radius; R, the particle radius; W, the force acting on the claw; P, contact point.

The theoretical model proposed by Dai et al. provides a framework for describing the frictional interaction between the claw tips and surface asperities on rough substrates [7]. The assumptions regarding the geometry of the substrate in this study were consistent with those of Dai et al., as shown in Fig 4.

Friction depends on the maximum resistance met by the claw as it slides over the particle surface. Sliding happens only when the angle α, representing the ratio of claw tip diameter to particle size, is large enough. Below a certain threshold, the substrate particles block the claw tip from sliding, promoting mechanical interlocking. In this scenario, the frictional force is solely determined by the force applied by the honey bee.

The extreme case is derived from equilibrium conditions. Based on the force balance condition in the direction perpendicular to the contact point, the contact force N is:


$$\begin{array}{c} \begin{array}{c} N=Fcos\alpha \end{array}+Wsin\alpha \; \end{array}$$ (1)

In the tangential direction at the contact point, we have:


$$\begin{array}{c} \begin{array}{c} Fsin\alpha =Wcos\alpha \end{array}+f\cdot N\; \end{array}$$ (2)

where *f* is friction coefficient between claw tip and particles.

Based on the equilibrium condition at the contact point, the relationship between *F/W*, contact angle *α*, and friction coefficient *f* can be determined using Equation (3) as follows:


$$\begin{array}{c} \begin{array}{c} \frac{F}{W}=\frac{cos\;\alpha \;+\;f\;sin\;\alpha }{sin\;\alpha \;-\;f\;cos\;\alpha }=\frac{1\;+\;f\;tan\;\alpha }{tan\;\alpha \;-\;f} \end{array}\; \end{array}$$ (3)

where *F/W* represents the ratio of the horizontal force to the normal force at the contact point, and f is the friction coefficient between the claw tip and particles. The contact angle *α* can be defined using Equation (4) as follows:


$$\begin{array}{c} \begin{array}{c} \sin\;\alpha =\frac{r+h}{r+R}=\frac{\frac{r}{R}+\frac{h}{R}}{\frac{r}{R}+1} \end{array} \end{array}\;$$ (4)

where h is the immersion depth of the particle, α represents the contact angle, r is the claw tip radius, and R represents the particle radius. Based on Equation (4), the deeper the particle is immersed in the substrate (the larger the value of h), or the smaller the particle radius R, the greater the contact angle α.

Moreover, based on Equation (3), when the contact angle α is sufficiently large, satisfying tan α−f > 0, the maximum value of the tangential force *F* can be calculated based on the normal force *W* applied by the bee’s claw tip (obtained from adhesion force measurements).

**Fig 5:**
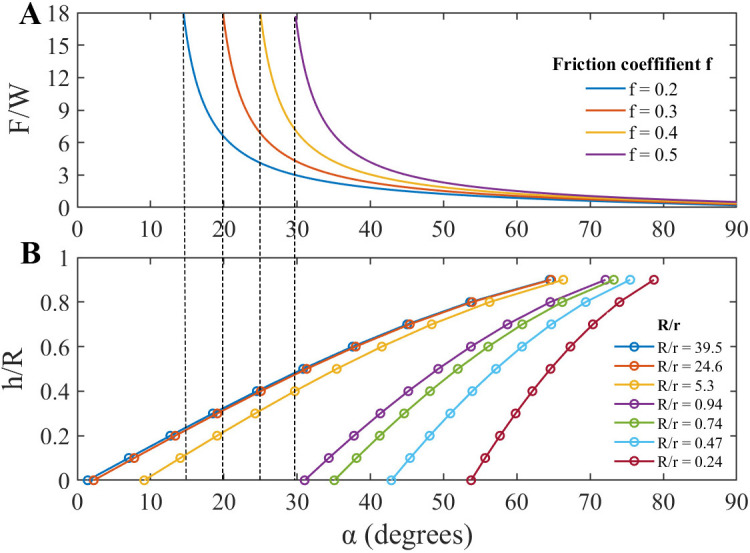
Conditions necessary for mechanical interlocking. **(A)** Dependence of the force ratio *F*/*W* (*F* represents the leg force, and *W* is the force acting on the claw) on the contact angle (*α*) at different friction coefficients between the claw and particles of the sandpaper. **(B)** Dependence of *h*/*R* (h represents the immersion depth of the particle, and R is the particle radius) on the contact angle (*α*) at different values of the relationship *R*/*r* (particle radius/ claw tip radius). When the diameter of a particle is comparable to the claw tip diameter (*R*/*r* = 1), both structures cannot interlock even at the friction coefficient f = 0.5. When *R*/*r* exceeds 5, the structures may interlock even at f* *= 0.2. The model predicts the relative maximum force depending on the friction coefficient at contact, the diameter of particles, and the immersion depth. Broken lines divide the ranges of *α* at which interlocking (self-locking) occurs (left side) at a particular friction coefficient value.

If the tangential force generated by the honey bee exceeds this limit, the claw will slip. Equation (3) effectively predicts the tangential force exerted by honey bees on surfaces with different roughness values. For example, as shown in Fig 5, on sandpaper with an average particle diameter of 6.4 μm (R/r=0.94), the minimum contact angle was 31.1°. When h = 0 and f =0.5, 0.4, 0.3, 0.2, the predicted forces were 7.29, 3.49, 2.21, and 1.58 mN, respectively. Among these, f =0.5 (7.29 mN) was close to the experimentally measured shear force (7.45 mN) applied by honey bees without adhesive pads on the surface with an average particle diameter of 6.4 μm. When the friction coefficient was 0.5, the contact friction between the claw tip and particles was moderate, ensuring sufficient contact stability while preventing excessive slipping (lower friction coefficients may lead to slipping). Under these conditions, the interlocking effect was more pronounced, and the friction generation mechanism was the most stable. Therefore, a comparative analysis of the predicted and experimental results at a friction coefficient of 0.5, effectively validated the accuracy of this model.

When the particle diameter was larger than the claw tip diameter, Equation (3) yielded a smaller contact angle α, resulting in tan α−f<0, which indicates that the claw tip and surface particles form a stable mechanical interlock, preventing the claw from slipping. Under this condition, the measured force reflects only the force produced by the bee’s leg. According to the model proposed by Dai et al., when the friction coefficient is as low as 0.2, the claw tip may not be strongly restricted by sliding resistance(7). Consequently, it can be embedded more deeply into the particle surface, enhancing mechanical interlocking. Under these conditions, on a substrate surface with an average particle size of 36 μm, the predicted maximum tangential force was 10.17 mN, slightly lower than the experimentally obtained 12.22 mN. In reality, the friction coefficient is likely greater than 0.2, and the particle immersion depth h is often greater than zero, which explains why the predicted values were slightly lower than the experimental results.

### Liquid bridge model of contact between adhesive pad and substrate surface

The adhesion force applied by the adhesive pad of a bee is mainly determined by the geometric characteristics of the liquid bridge formed between the adhesive pad and the substrate surface through mucus secretion. We established a liquid bridge model of contact between the substrate and adhesive pad to explore the variations in adhesion force applied by the pad under varying surface roughness, quantify the effects of the liquid bridge on this force, and investigate the relation between the geometry of the bridge and surface roughness. To construct this model, the following assumptions were made:

(1) Liquid bridge-filling assumption: On smooth surfaces or surfaces with low roughness, mucus can fill the microscopic gaps of the substrate; on surfaces with high roughness, owing to increased surface irregularity, mucus cannot fill the microscopic gaps.
(2) Normal force model assumption: The mucus secreted by the adhesive pad surface uniformly covers the entire surface of the adhesive pad. Under the action of a liquid bridge, both the capillary and viscous forces contribute to the normal force.

The adhesive force of the liquid bridge model originates from the capillary and viscous forces of the liquid. On surfaces with different roughness, the state of the liquid bridge is mainly divided into two types: one is when the liquid can cover the gap between the two planes, typically occurring on smoother surfaces or surfaces with lower roughness, and the other is when the liquid cannot cover the gap between the two planes, which is more common on surfaces with higher roughness. This study focused on exploring the contribution of the liquid in these two states to the adhesive force.

**Fig 6:**
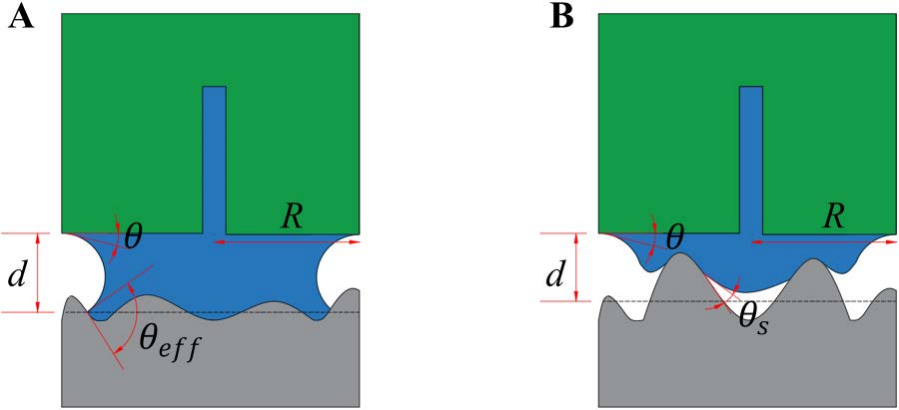
Distribution of fluid bridges between the foot pads of bees and the contact planes. The green graphic represents the adhesion pad, the gray part is the substrate surface and the blue part is the liquid bridge. **(A)** Mucus secreted by the foot pads fills the gaps between the rough bodies. **(B)** Mucus secreted by the footpad does not fill the gaps between the rough bodies. *d*, the height of the liquid bridge; θ, the contact angle of the smooth plane; *R*, the radius of the liquid bridge; $\theta_{eff}$, the effective contact angle; and $\theta_{s}$, the cape of suspension.


When the liquid filled the gap between the two planes (Fig 6A), the capillary force mainly originated from the surface tension of the liquid. The surface tension of the liquid forms a liquid bridge between solid surfaces, thereby generating adhesion. For a liquid bridge with a radius *R*, the capillary force $F_{cap}$ can be expressed using Equation (5) as follows [17,18]:

$$\begin{array}{c} \begin{array}{c} F_{cap}=2\pi R\gamma_{LV}\;cos{\;\theta }_{eff} \end{array} \end{array}$$ (5)

where $\gamma_{LV}$ is the liquid–gas interfacial tension, and $\theta_{eff}$ is the effective contact angle of the liquid on a rough surface, reflecting the modification of the contact angle because of the surface roughness structure.

The surface structure alters the contact angle of the liquid on rough surfaces. This phenomenon can be described using the Wenzel model [19]. The relationship between the effective contact angle $\theta_{eff}$ on a rough surface and the contact angle θ on a smooth surface can be expressed using Equation (6) as follows:


$$\begin{array}{c} \begin{array}{c} cos\;{\;\theta }_{eff}=r\;cos\;\theta \end{array} \end{array}$$ (6)

where r is the roughness factor, defined as the ratio of the actual surface area of the rough surface to its projected area, and θ is the contact angle on a smooth surface.

During surface separation, the viscous force of the liquid arises from the resistance of the liquid viscosity to the separation motion. The magnitude of the viscous force $F_{vis}$ is related to the viscosity of the liquid, effective contact area, height of the liquid bridge, and separation velocity, and can be expressed using Equation (7) as follows [20]:


$$\begin{array}{c} \begin{array}{c} F_{vis}=\eta \frac{A_{eff}}{d}\frac{dh}{dt} \end{array} \end{array}$$ (7)

where η is the dynamic viscosity of the liquid, $A_{eff}$ represents the actual contact area between the liquid and the substrate, d is the height of the liquid bridge, and $\frac{dh}{dt}$ represents the detachment velocity between the adhesive pad and the substrate, and is considered to be independent of the liquid bridge height.

When a liquid fills the pits on the substrate surface, the actual contact area of the liquid, $A_{eff}$, is typically larger than that of the ideal surface. It can be expressed as $A_{eff}\;=\;Ar$, where 1≤r. Thus, under these conditions, $F_{vis}$ can be expressed using Equation (8) as follows:


$$\begin{array}{c} \begin{array}{c} F_{vis}=\eta \frac{A\;r}{d}\frac{dh}{dt} \end{array} \end{array}$$ (8)

The total adhesion force *F* can be expressed using Equation (9) as follows:


$$\begin{array}{c} \begin{array}{c} F=F_{cap}+F_{vis} \end{array} \end{array}$$ (9)

Thus, the height of the liquid bridge can be calculated using Equation (10) as follows:


$$\begin{array}{c} \begin{array}{c} d=\frac{\eta Ar\frac{dh}{dt}}{F-2\pi R\gamma_{LV}r\cos\theta } \end{array} \end{array}$$ (10)

In this study, the liquid bridge height is assumed to be determined by the contact area A, the roughness factor r, and the contact angle θ.

When the liquid does not fill the gap between the two planes (Fig 6B), the liquid bridge may exist in a suspended form, potentially forming a partial contact or a suspended liquid bridge on a rough surface. In this scenario, the surface tension of the liquid causes the shape of the liquid bridge to curve, forming a locally suspended liquid bridge that typically resembles a curved arc or spherical cap [21,22]. The effective contact angle, $\theta_{eff}$, may no longer be applicable or need to be considered, as the liquid does not fill the space between the planes and the substrate surface, and it may not be in direct contact with the substrate surface. Thus, the effects of roughness on the contact angle can be neglected.

The suspended liquid bridge forms a suspended angle $\theta_{s}$ at the curved part, the angle formed by the liquid after being released from the plane and suspended on the rough surface. The capillary force at this point can be adjusted by introducing a correction factor for the suspended angle ${cos\theta }_{s}$ [23] and can be expressed using Equation (11) as follows:


$$\begin{array}{c} \begin{array}{c} F_{cap}=2\pi R\gamma_{LV}\;\cos\;{\theta \;cos\;\theta }_{s} \end{array} \end{array}$$ (11)

When $\theta_{s}$ is large, the liquid curvature is relatively more pronounced, and the range of capillary force distribution increases, whereas, when $\theta_{s}$ is small, the liquid curvature is less, and the capillary force is more concentrated in the contact area of the smooth plane.

At this point, the actual contact area $A_{eff}$ is typically smaller than that of the ideal surface, and $A_{eff}$ on the rough surface can be expressed using a scaling factor, *f*, i.e., $A_{eff}=A\;n$, where n is the scaling factor for the contact area and 0<n≤1.

Thus, under these conditions, the height of the liquid bridge can be calculated using Equation (12) as follows:


$$\begin{array}{c} \begin{array}{c} d=\frac{\eta An\frac{dh}{dt}}{F-2\pi R\gamma_{LV}\cos\theta {cos\theta }_{s}} \end{array} \end{array}$$ (12)

**Fig 7:**
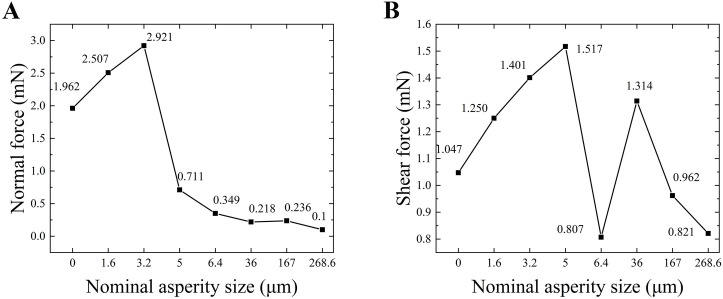
Measurements of the normal and shear forces applied when the adhesive pad of a bee acts alone. **(A)** Measurement of normal force when the adhesive pad acts alone. **(B)** Measurement of shear force when the adhesive pad acts alone.

The average difference between the forces measured on the substrate surface for the complete honey bee and the honey bee without the adhesive pad (Fig 7) was considered the adhesive force generated solely by the adhesive pad on the substrate surface. As shown in Fig 7A, On surfaces with roughness from 0 to 3.2 μm, the adhesive liquid fills the gap between the adhesive pad and the substrate. As roughness increases, the contact area between the adhesive pad’s liquid and the substrate grows. Since adhesive force is proportional to contact area (as shown in Equation (7)), the adhesive force increases when only the adhesive pad is acting. However, on surfaces with roughness of 5 μm or more, the liquid cannot fill this gap, leading to a reduction in contact area and a consequent decrease in adhesion.

The measured normal adhesive force values from the experiments were substituted into the liquid bridge model for analysis, and the other relevant parameter values are listed in Table 3. A few parameter values were obtained from literature [6].

**Table 3:** Parameter values used for the liquid bridge.

Parameter	γ	η	dh/dt	R	A	θ	$\theta_{s}$
**Value**	28 mN/m	100 mPa/s	5 mm/s	100 μm	$200\times 200\;{\mu m}^{2}$	π/6	π/6

^a^γ, liquid–gas interfacial tension, η, the dynamic viscosity of the liquid; *dh/dt*, separation velocity; *R*, the radius of the liquid bridge; A is the actual contact area between the liquid and the substrate; θ, the contact angle of the smooth plane; and $\theta_{s}$, the cape of suspension.

For the validation of the model, the suspension angle $\theta_{s}$ was selected to be 30° as a representative value. When the suspension angle varied between 0° and 90°, the capillary force slightly decreased with the increase in suspension angle, with a change range of ≤0.01 mN. Therefore, the effects of the suspension angle on the adhesive force were negligible.

**Fig 8:**
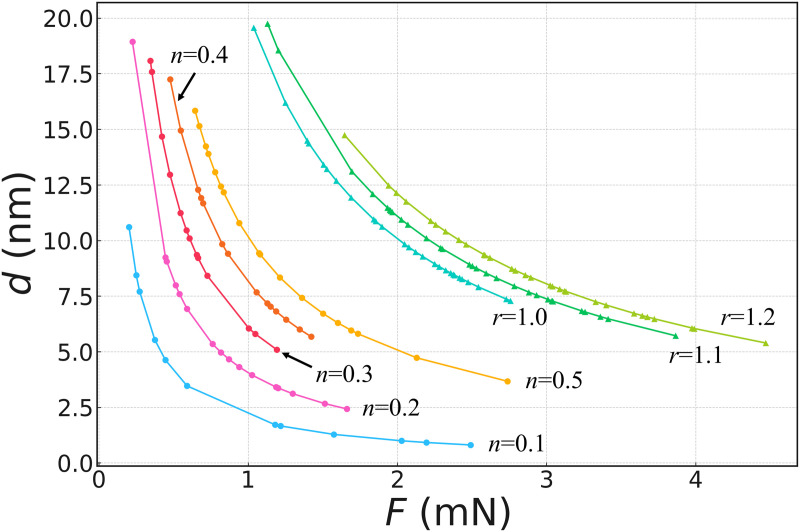
Relationship between normal adhesion force F and liquid bridge height d. *n,* the scaling factor for the contact area; *r*, the roughness factor.

Fig 8 shows the relationship between the normal force F and the liquid bridge height d obtained by substituting the experimentally measured normal force into the liquid bridge model. This includes two typical scenarios: r = 1.0, 1.1, and 1.2, indicating the case in which the liquid fills the gap between the two surfaces, and n = 0.5, 0.4, 0.3, 0.2, and 0.1, indicating the case in which the liquid cannot fill the gap. The curves revealed the different mechanisms and relative contributions of the liquid bridge height (d), roughness factor (r), and contact area ratio factor (n) in regulating the capillary and viscous forces.

As the roughness factor *r* increased, the effective contact area increased, enhancing the contributions of the capillary and viscous forces. The experiment revealed that the roughness factor regulated the enhancement of the capillary force by impacting the contact angle and contact area and significantly amplified the role of viscous forces. Furthermore, a reduction in the height of the liquid bridge significantly increased the viscous force. When the liquid bridge height decreased below 10 nm, the effect of the viscous force rapidly increased, indicating that changes in the liquid bridge height played a key role in enhancing the normal force. This trend is consistent with the study by Pitois et al. [24], who pointed out that, in the case of viscous liquid bridges, when the separation distance decreases, the viscous force increases significantly because of the reduction in the liquid bridge height and the increase in the velocity gradient within the liquid bridge.

As the scaling factor n for the contact area decreased from 0.5 to 0.1, the effective contact area gradually decreased, weakening the combined effects of the capillary and viscous forces, which is reflected in the decrease in the normal force. When n=0.1, and the liquid bridge height dropped below 15 nm, the increase in the viscous force partially compensated for the adverse effects caused by a reduction in the contact area. Owing to the scaling effects of height and volume, a decrease in the liquid bridge volume leads to a significant increase in the viscous force.

By comparing the liquid bridge height derived from the experiment with that produced by other insects, it was found that the liquid bridge height derived in this study was consistent with the typical liquid bridge heights reported in the literature for insects (less than 10–20 nm) [25].

For the shear force, in addition to the shear capillary and viscous forces generated by the liquid, the dry friction between the adhesive pad and contact surface is also a critical factor that cannot be ignored. The dry friction effect refers to the friction generated when adhesive pads are in direct contact with the substrate, which is positively correlated with their contact area. The greater the contact area, the stronger the dry friction effect. According to the experimental results presented in Fig 7B, the trend of the shear force when the adhesive pad acted alone differed significantly from that of the normal force. This is primarily determined by the combined effects of the liquid bridge and dry friction. On surfaces with roughness values less than 3.2 μm, the liquid can fully fill the gap between the adhesive pad and the substrate surface, significantly enhancing both the adhesive and shear forces, following a trend similar to that of the normal force. On surfaces with a roughness value of 5 μm, although the liquid bridge effect weakens, the dry friction effect reaches its peak due to the increased contact area, maximizing the shear force. When the roughness increases further to 6.4 μm, the liquid bridge effect is significantly weakened, and the dry friction effect, due to the decrease in contact area, is insufficient to compensate for the weakened liquid bridge, causing the shear force to drop sharply. As the roughness increases to 36 μm, the friction coefficient increases, causing the shear force to rise again. In the higher roughness range (167–268.6 μm), because the adhesive pad cannot fully adapt to the shape of the rough surface, the interlocking between the pad and the particles on the substrate weakens, leading to a further decrease in shear force [26]. The experimental data are consistent with the findings in the literature, indicating that the variation in shear force is the result of both the liquid bridge and dry friction effects, with the dominance of each varying significantly depending on the contact conditions at different roughness levels.

## Conclusion

This study provides a comprehensive understanding of the dual-mode synergistic adhesion mechanism in honey bees on surfaces with varying roughness levels. Our findings reveal that the adhesive performance of honey bees is significantly influenced by the interplay between mechanical interlocking (via claws) and liquid bridge formation (via adhesive pads). The key conclusions of our study are as follows:

1. Distinct Roles of Claws and Adhesive Pads: On surfaces with low roughness (≤ 3.2 μm), adhesive pads dominate the adhesion process by secreting liquid to form liquid bridges, which increase contact area and adhesive force. As surface roughness increases, the contribution of adhesive pads diminishes, while the mechanical interlocking effect of claws becomes more pronounced. On surfaces with high roughness (≥ 36 μm), the irregularity of substrate particles enhances the interlocking effect of claws, making the force generated by the claw tip the dominant adhesion force.
2. Synergistic Effects: The integration of contact and liquid bridge models demonstrates the synergistic effects of mechanical interlocking and liquid bridge mechanisms on surface adhesion. This dual-mode mechanism allows honey bees to adapt effectively to a wide range of surface roughness conditions.
3. Experimental Validation: Our experimental results validate the applicability of the mechanical interlocking and liquid bridge models for analyzing adhesion performance on surfaces with different roughness levels. The findings provide valuable insights into the adhesion mechanisms of honey bees and offer a theoretical basis for the design of bioinspired adhesives and micro-climbing robots.
4. Implications for Biomimetic Design: The study highlights the importance of considering both mechanical interlocking and liquid bridge formation in the design of bioinspired adhesives. By mimicking the dual-mode adhesion mechanism of honey bees, it is possible to develop adhesives that can effectively adhere to surfaces with varying roughness levels, enhancing their practical applications in diverse environments.

## Supporting information

S1 File Data on the normal and shear adhesive forces measured for intact bees and bees with adhesive pads removed on surfaces with different roughness levels. (XLSX)

## References

[1] Bullock JMR, Drechsler P, Federle W. Comparison of smooth and hairy attachment pads in insects: friction, adhesion and mechanisms for direction-dependence. J Exp Biol. 2008;211(Pt 20):3333–43. doi: 10.1242/jeb.020941 18840668

[2] Federle W, Brainerd EL, McMahon TA, Holldobler B. Biomechanics of the movable pretarsal adhesive organ in ants and bees. Proc Natl Acad Sci U S A. 2001;98(11):6215–20. doi: 10.1073/pnas.111139298 11353847 PMC33448

[3] Dirks J, Federle W. Fluid-based adhesion in insects–principles and challenges. Soft Matter. 2011;7(23):11047–53. 10.1039/c1sm06269g 41776880

[4] Daltorio KA, Boxerbaum AS, Horchler AD, Shaw KM, Chiel HJ, Quinn RD. Efficient worm-like locomotion: slip and control of soft-bodied peristaltic robots. Bioinspir Biomim. 2013;8(3):035003. doi: 10.1088/1748-3182/8/3/035003 23981561

[5] Endlein T, Federle W. Walking on smooth or rough ground: passive control of pretarsal attachment in ants. J Comp Physiol A Neuroethol Sens Neural Behav Physiol. 2008;194(1):49–60. doi: 10.1007/s00359-007-0287-x 18060411

[6] Labonte D, Federle W. Rate-dependence of “wet” biological adhesives and the function of the pad secretion in insects. Soft Matter. 2015;11(44):8661–73. doi: 10.1039/c5sm01496d 26376599

[7] Dai Z, Gorb SN, Schwarz U. Roughness-dependent friction force of the tarsal claw system in the beetle Pachnoda marginata (Coleoptera, Scarabaeidae). J Exp Biol. 2002;205(Pt 16):2479–88. doi: 10.1242/jeb.205.16.2479 12124371

[8] Persson BNJ, Gorb S. The effect of surface roughness on the adhesion of elastic plates with application to biological systems. J Chem Phy. 2003;119(21):11437–44. doi: 10.1063/1.1621854

[9] Prokkola JM, Nikinmaa M, Lewis M, Anttila K, Kanerva M, Ikkala K, et al. Cold temperature represses daily rhythms in the liver transcriptome of a stenothermal teleost under decreasing day length. J Exp Biol. 2018;221(Pt 5):jeb170670. doi: 10.1242/jeb.170670 29361589

[10] Chen J, Wang P, Li M, Shen J, Howes T, Wang G. Rupture distance and shape of the liquid bridge with rough surface. Minerals Eng. 2021;167:106888. doi: 10.1016/j.mineng.2021.106888

[11] BakhshandehSeraji R, Palasantzas G. Nanoscale-roughness influence on pull-off adhesion force in liquid and air. Phys Rev E. 2023;108(5–1):054801. doi: 10.1103/PhysRevE.108.054801 38115441

[12] Nguyen HNG, Zhao C, Millet O, Selvadurai A. Effects of surface roughness on liquid bridge capillarity and droplet wetting. Powder Technol. 2021;378:487–96.

[13] Müser MH, Nicola L. Modeling the surface topography dependence of friction, adhesion, and contact compliance. MRS Bull. 2022;47(12):1221–8. doi: 10.1557/s43577-022-00468-2 36846502 PMC9947065

[14] Zhu Z, Gu Y, Liang L, Zhao J, Yu Z. Simulation analysis on surface morphology and superior adhesive properties of honeybee footpad. J Adhes Sci Technol. 2023;37(14):2069–81.

[15] Federle W, Baumgartner W, Hölldobler B. Biomechanics of ant adhesive pads: frictional forces are rate- and temperature-dependent. J Exp Biol. 2004;207(Pt 1):67–74. doi: 10.1242/jeb.00716 14638834

[16] Dang Nguyen D, Phuong Dam N, Anh Ho V. Enhancing wet surface adhesion in walking robots: finite element-based analysis and morphology-changeable soft pads. Adv Robotics. 2024;38(8):524–40.

[17] Israelachvili JN. Intermolecular and surface forces. Academic Press; 2011.

[18] Mason G, Clark WC. Liquid bridges between spheres. Chemical Eng Sci. 1965;20(10):859–66. doi: 10.1016/0009-2509(65)80082-3

[19] Wenzel RN. Resistance of solid surfaces to wetting by water. Industr Eng Chem. 1936;28(8):988–94.

[20] Derjaguin BV, Churaev NV, Muller VM. Wetting films. Surface Forces. 1987:327-–67.

[21] Alam M, Saha S, Gupta R. Unified theory for a sheared gas–solid suspension: from rapid granular suspension to its small-Stokes-number limit. J Fluid Mech. 2019;870:1175–93. doi: 10.1017/jfm.2019.304

[22] Lain S, Sommerfeld M. Influence of droplet collision modelling in Euler/Lagrange calculations of spray evolution. Inter J Multiphase Flow. 2020;132:103392. doi: 10.1016/j.ijmultiphaseflow.2020.103392

[23] Gennes P, Brochard-Wyart F, Quéré D. Capillarity and wetting phenomena: drops, bubbles, pearls, waves. Springer; 2004.

[24] Pitois O, Moucheront P, Chateau X. Liquid bridge between two moving spheres: an experimental study of viscosity effects. J Colloid Interface Sci. 2000;231(1):26–31. doi: 10.1006/jcis.2000.7096 11082244

[25] Hosoda N, Nakamoto M, Suga T, Gorb SN. Evidence for intermolecular forces involved in ladybird beetle tarsal setae adhesion. Sci Rep. 2021;11(1):7729. doi: 10.1038/s41598-021-87383-9 33833354 PMC8032735

[26] Persson BNJ, Scaraggi M. Theory of adhesion: role of surface roughness. J Chem Phys. 2014;141(12):124701. doi: 10.1063/1.4895789 25273455

